# Summer precipitation anomalies in Asia and North America induced by Eurasian non-monsoon land heating versus ENSO

**DOI:** 10.1038/srep21346

**Published:** 2016-02-26

**Authors:** Ping Zhao, Bin Wang, Jiping Liu, Xiuji Zhou, Junming Chen, Sulan Nan, Ge Liu, Dong Xiao

**Affiliations:** 1 State Key Laboratory of Severe Weather, Chinese Academy of Meteorological Sciences, Beijing, China; 2 Collaborative Innovation Center on Forecast and Evaluation of Meteorological Disasters, Nanjing University of Information Science and Technology, Nanjing, China; 3 International Pacific Research Center, School of Ocean and Earth Science and Technology, University of Hawaii at Manoa, Honolulu, HI, USA; 4 Earth System Modeling Center, Nanjing University of Information Science and Technology, Nanjing, China; 5 Department of Atmospheric and Environmental Sciences, University at Albany, State University of New York, Albany, NY, USA

## Abstract

When floods ravage Asian monsoon regions in summer, megadroughts often attack extratropical North America, which feature an intercontinental contrasting precipitation anomaly between Asia and North America. However, the characteristics of the contrasting Asian-North American (CANA) precipitation anomalies and associated mechanisms have not been investigated specifically. In this article, we firmly establish this summer CANA pattern, providing evidence for a significant effect of the land surface thermal forcing over Eurasian non-monsoon regions on the CANA precipitation anomalies by observations and numerical experiments. We show that the origin of the CANA precipitation anomalies and associated anomalous anticyclones over the subtropical North Pacific and Atlantic has a deeper root in Eurasian non-monsoon land surface heating than in North American land surface heating. The ocean forcing from the ENSO is secondary and tends to be confined in the tropics. Our results have strong implications to interpretation of the feedback of global warming on hydrological cycle over Asia and North America. Under the projected global warming due to the anthropogenic forcing, the prominent surface warming over Eurasian non-monsoon regions is a robust feature which, through the mechanism discussed here, would favor a precipitation increase over Asian monsoon regions and a precipitation decrease over extratropical North America.

The variations of precipitation over the northern hemispheric monsoon regions and the adjacent arid regions have been studied using observations and model simulations^1^,^2^,^3^,^4^. There is evidence that when floods occur in Asian monsoon regions, megadroughts often concur in extratropical North America. In summer 2012, when extratropical North America was attacked by the severest drought, East Asia was ravaged by the heaviest rainfall (Fig. S1a) that was directly responsible for 80 deaths in Beijing. Under the CMIP5 RCP 4.5 scenario, summer precipitation will increase over Asia, whereas it will decrease over North America^5^ (Fig. S1b). These suggest a possible intercontinental contrasting CANA precipitation anomaly pattern. However, its characteristics and associated mechanisms have not been investigated specifically. Ocean-atmosphere and land-atmosphere interactions are often considered as important factors in regulating global and regional climate^6^,^7^,^8^,^9^,^10^,^11^,^12^,^13^,^14^,^15^; especially the influences of land-atmosphere interactions in Asian and African monsoon regions have extensively been investigated^9^,^11^,^13^,^14^,^15^ and an increased surface temperature over Eurasia modifies the relationship between the ENSO and monsoon^16^,^17^. Here we examine the robustness of the summer CANA precipitation teleconnection by analyzing 100-year observational rainfall data, and show that land surface heating over Eurasian non-monsoon regions (Europe and West Asia) is a more dominant factor influencing this precipitation teleconnection compared to the North American land surface heating anomaly and the ocean forcing from the ENSO by conducting a suite of numerical experiments.

## Results

**Figure 1:**
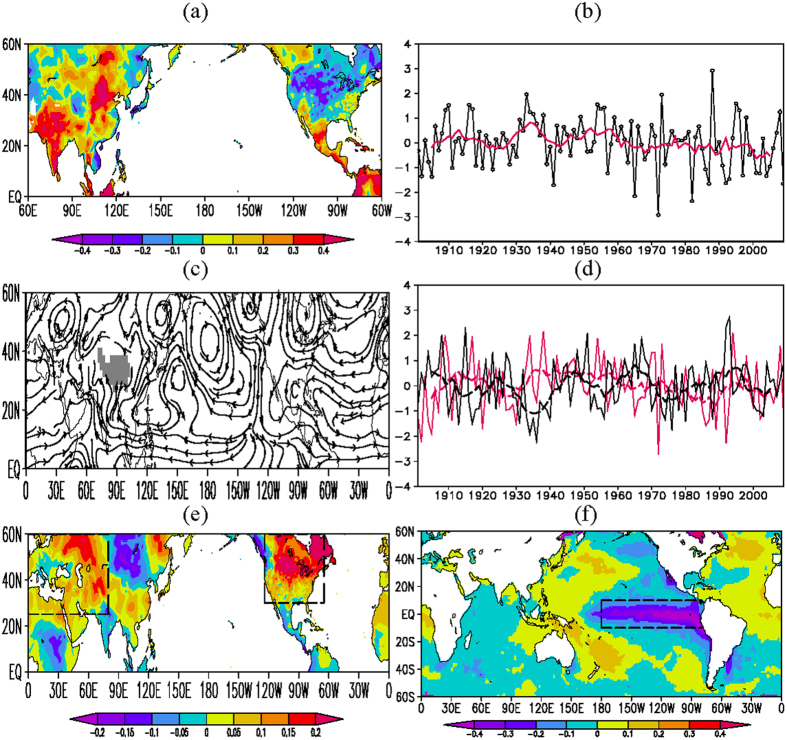
(**a**) JJA mean CRU precipitation EOF1 mode (PC1) during 1901–2009; (**b**) the standardized time series of PC1 (black) and its nine-year running mean value (red); (**c**) regression of JJA mean 700-hPa flow of the twentieth century reanalysis V2 products against the PC1 during 1901–2009, in which the grey shaded area is for topography; (**d**) the standardized time series of JJA mean precipitation over Asia (the red solid line) and North America (115°W-95°W, 35°N-45°N; the black solid line) during 1901–2009 and their nine-year running means (dashed lines); (**e**) regression of AMJJA mean CRU surface air temperature against the PC1 during 1901–2009, in which two boxes indicate the subtropical and midlatitude Eurasia and the mid- and high-latitude North America, respectively; and (**f**) same as in (**e**) but for HadISST SST, in which the box indicates the equatorial central-eastern Pacific. These figures are generated by Grid Analysis and Display System (GrADS) Version 2.0.1.oga.1 with Copyright (**c**) 1988–2011 by Brian Doty and the Institute for Global Environment and Society (IGES) (ftp://cola.gmu.edu/grads/2.0/old/).

We begin with presenting firm evidence for the existence of the intercontinental contrasting CANA precipitation anomaly pattern. The Empirical Orthogonal Function (EOF) analysis on the summer (June-August, JJA) mean standardized precipitation anomaly of the Climatic Research Unit (CRU) analysis in the region 60°E-60°W and 0°-60°N during 1901-2009 reveals that the first EOF mode (EOF1, significant at the 98% confidence level through 1000-time Monte Carlo simulations), accounting for 5% of the total variance, is characterized by positive precipitation anomalies in large areas of South and East Asia and central-eastern Russia and in the tropics of South and North America, with the centers over the Indian Peninsula and the middle latitudes of East Asia, and negative precipitation anomalies in North America between 35°N and 50°N, with the center over the Great Plain of North America (Fig. 1a). The leading principal component (PC1) has a positive correlation (r = 0.63) with the JJA mean precipitation averaged over Asia (70°E-120°E, 10°N-60°N) and a negative correlation (r = −0.42/−0.35) with the precipitation averaged over the Great Plains (115°W-95°W, 35°N-45°N)/the mid and high latitudes (120°W-60°W, 30°N-60°N) of North America (>99.9% confidence level) (Fig. 1b,d). Meanwhile, there is a significant negative correlation between the Asian and North American precipitation time series, and it is generally stable for the past 100 years (Fig. S1c). The results indicate that the out-of-phase relationship in the precipitation between Asia and North America (the CANA pattern) represents a predominant feature of the summer precipitation variability between Asia and North America.

Associated with the CANA pattern are lower-tropospheric anomalies that feature a cyclonic circulation anomaly over the middle and low latitudes of Asia and the tropics of North Africa and anticyclonic anomalies over the East Asian coasts, the North Pacific, and the North Atlantic between 20°N and 55°N (Fig. 1c). The lower-tropospheric southerly wind anomalies generally prevail over South Asia, East Asia, and central-eastern Russia, strengthening water vapor transport (Fig. S2) and increasing precipitation in Asian monsoon regions. The anticyclonic circulation anomaly over the extratropical North Atlantic stretches westward into extratropical North America, which suppresses precipitation over extratropical North America, and a cyclonic anomaly covers the tropics of South and North America.

Corresponding to a positive phase of the CANA precipitation pattern, the CRU surface air temperature (SAT) generally increases over Europe and West Asia from April to August (Fig. S3). Over North America, SAT mainly shows pronounced increases in April and from June to August. On the spring-summer (April-August, AMJJA) mean map (Fig. 1e), positive anomalies of SAT appear in the large areas of Europe, West Asia, and extratropical North America. There are significant correlations between the time series of the CANA precipitation pattern and the regional mean SAT over extratropical Eurasia (20°E-80°E, 30°N-60°N) or North America (120°W-60°W, 30°N-60°N) during 1901–2009 (>95% confidence level). It is evident that compared to global monsoon regions^4^,^5^, large positive SAT anomalies mainly appear in the non-monsoon regions (Europe, West Asia, and extratropical North America). The precipitation variability in the South Asian monsoon region, the midlatitudes of East Asia, central-eastern Russia, and the North American tropical monsoon region tends to be negatively correlated with local SAT. Such a negative correlation between land precipitation and SAT in summer has been noted by the previous studies^18^,^19^,^20^. This association between the CANA and SAT is also successfully captured by the National Center for Atmospheric Research (NCAR) Parallel Climate Model (PCM) 20C3M simulation for the period 1901 to 1999 (Fig. S4).

**Figure 2:**
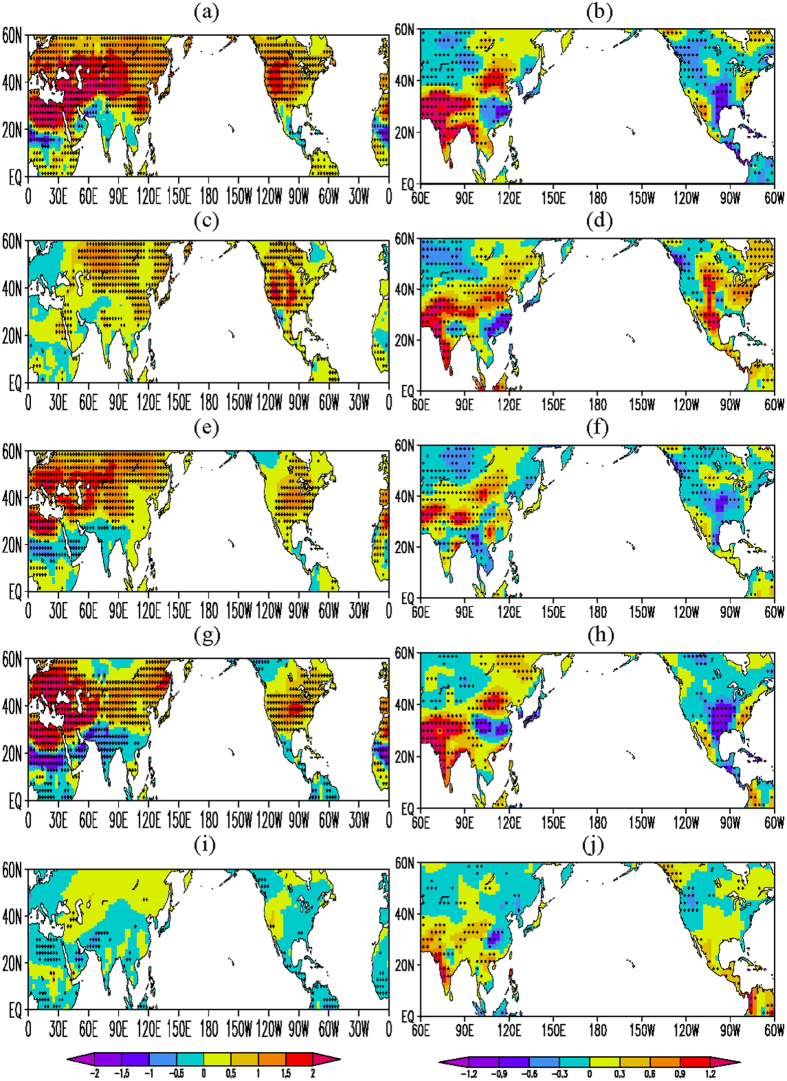
Responses of climate to the forcing of land surface heating and/or equatorial central-eastern Pacific cooling. For (**a**) AMJJA mean surface air temperature (°C) and (**b**) JJA mean precipitation (mm/day) anomalies from the Eurasian land surface thermal forcing in the CCSM3 model (CCSM3_EA minus CCSM3_C); (**c,d**) same as in (**a,b**) but for the North American land surface thermal forcing; (**e,f**) same as in (**a,b**) but in the CAM3 model with prescribed climatological mean SST; (**g,h**) same as in (**a,b**) but for the collaborative forcing of both Eurasian land surface heating and equatorial central-eastern Pacific cooling; and (**i,j**) same as in (**a,b**) but for the equatorial central-eastern Pacific forcing alone (CCSM3_C minus CCSM3_P). Black dots are at the 90% confidence level. These figures are generated by Grid Analysis and Display System (GrADS) Version 2.0.1.oga.1 with Copyright (c) 1988-2011 by Brian Doty and the Institute for Global Environment and Society (IGES) (ftp://cola.gmu.edu/grads/2.0/old/).

Because an increase of SAT may be due to a general enhancement of local surface sensible heat flux anomalies, here we test a hypothesis that the observed CANA precipitation and positive SAT anomalies over Eurasia and North America may arise partially from the land heating forcing over Eurasian or/and North American non-monsoon regions. Following the previous studies^9^,^13^,^21^, we force the NCAR’s Community Climate System Model version 3 (CCSM3, a coupled atmosphere-ocean model) by reducing AMJJA surface vegetation albedo in the Eurasian or/and North American non-monsoon region (see Methods for details). Corresponding to the decrease of Eurasian land surface albedo, there are significantly increased sensible heat fluxes at the surface over Europe and West Asia (Fig. S5a). Meanwhile, the latent heat flux remarkably increases over the northern edges of African and Asian monsoon regions, and generally decreases over Europe and West Asia (Fig. S5b). Compared to the sensible heat flux, however, the positive anomalies of latent heat flux are weaker. This indicates that the anomalous surface heating is mainly caused by the sensible heat flux. Associated with the increased sensible heat flux, large-scale positive anomalies of AMJJA mean SAT appear in the middle and high latitudes of Eurasia and North America, with the positive anomalous centers over northern Africa and East Europe and West Asia, and the negative anomalies appear over the African and South Asian monsoon regions (Fig. 2a). The warming over extratropical North America is associated with a decrease of precipitation (see the following paragraph). The positive anomalies over Europe, West Asia, the subtropics of East Asia, and North America are generally similar to the observation (Fig. 1e). This demonstrates that the observed surface warming in these regions as well as the cooling in the tropical monsoon regions of Africa, South Asia, and North America can be forced by the land surface heating anomaly in Europe and West Asia.

**Figure 3:**
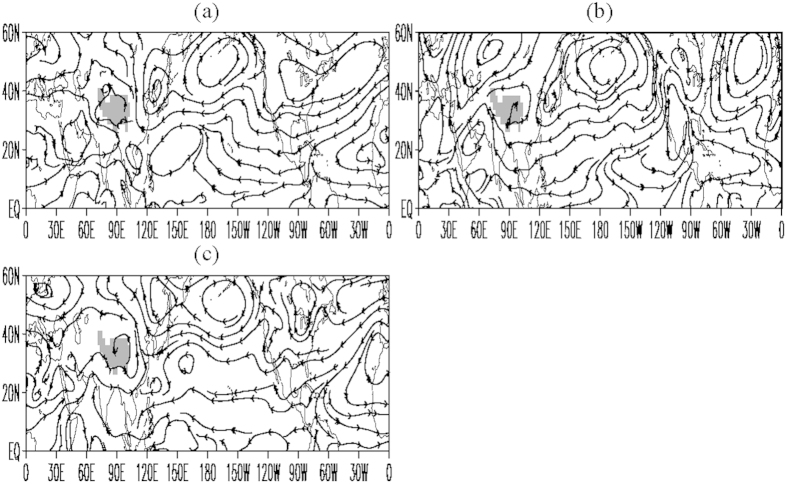
Responses of JJA mean 700-hPa flow to the forcing. (**a**) For the Eurasian land surface thermal forcing in the CCSM3 model; (**b**) same as in (**a**) but for the North American land surface thermal forcing; and (**c**) same as in (**a**) but in the CAM3 model with prescribed climatological mean SST. These figures are generated by Grid Analysis and Display System (GrADS) Version 2.0.1.oga.1 with Copyright (**c**) 1988-2011 by Brian Doty and the Institute for Global Environment and Society (IGES) (ftp://cola.gmu.edu/grads/2.0/old/).

Corresponding to this non-monsoon surface heating anomaly, large-scale positive precipitation anomalies occur over the Indian Peninsula and the mid latitudes of East Asia, while large-scale negative precipitation anomalies appear in North America between 30°N and 60°N (Fig. 2b). This forced anomaly feature generally resembles the CANA precipitation pattern. In the lower troposphere, cyclonic anomalies appear over Asia and Africa, and anticyclonic anomalies appear over the East Asian coasts, the North Pacific, and the Atlantic between 30°N and 55°N (Fig. 3a). The southerly wind anomalies generally prevail over South Asia, East Asia, and central-eastern Russia and the anticyclonic anomalies over the Atlantic stretches westward into extratropical North America. These simulated circulation anomaly features show good agreements with the observation. The consistency demonstrates that the observed anomalies in SAT, air flow, and precipitation may be a response to the surface heating anomaly in Europe and West Asia.

The question is how the Europe-West Asia land surface heating affects the atmospheric circulation over North America that is responsible for the local reduced rainfall. To give an explanation for this, we firstly examine a response of tropospheric temperature to the Eurasian land surface heating anomaly. Climatologically, positive values of the eddy temperature (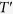, the departure from zonal mean) generally appear in the entire troposphere over this region, and a large-scale anticyclonic circulation covers Eurasia, Africa, and the western Pacific to the south of 50°N in the upper troposphere (namely the South Asian high) (Fig. S6). When the Eurasian land surface heating increases, a large-scale positive temperature anomaly appears in the troposphere over Eurasia (Fig. S7a), indicating a stronger than normal tropospheric temperature. Meanwhile, large-scale positive/negative eddy geopotential height (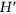) anomalies appear in the middle-upper/lower troposphere over Eurasia (Fig. S7a), indicating a stronger than normal South Asian high/Asian lower-tropospheric low (Fig. S6a). Some studies have shown that in response to a land heating anomaly over the Eurasian land, a classic conceptual model is characterized by the local ascent anomalies (resulting from the local lower-tropospheric low and upper-tropospheric high anomalies) and descent anomalies both to the east and to the west of the ascent anomalies, especially to the west^13^,^22^. In this study, the tropospheric ascent anomalies between 30°E and 80°E are forced by the Europe-West Asia land heating anomaly, with descent anomalies to the west and east of the ascent anomalies (Fig. S8a). Descent anomalies generally appear over a large area from the western Pacific to the Atlantic, with positive 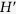 anomalies in the mid-lower troposphere over this area (Fig. S7a). These responses of temperature, geopotential height, and vertical motion to the Eurasian land surface heating anomaly are generally similar to the observations, although there are some differences between 150°W and 105°W (Fig. S8c). This also suggests a more dominant effect of the land surface heating anomalies on the west of the ascent anomalies, supporting the previous result^19^. Thus the above mechanism explains the effect of the Europe-West Asia land surface heating anomaly on the atmospheric circulation over extratropical North America.

Relative to the Eurasian heating anomaly, the surface heating anomaly over the North American non-monsoon region does not exert a similar impact. Strong positive temperature anomalies appear at the surface (Fig. 2c) and in the entire troposphere over North America, with strong high/low pressure anomalies in the upper/lower troposphere (Fig. S7b), and southerly wind anomalies generally appear over central-eastern North America (Fig. 3b). Accordingly, strong tropospheric ascent anomalies (Fig. S8b) and positive precipitation anomalies (Fig. 2d) occur over central-eastern North America. Meanwhile, positive precipitation anomalies also appear in the Asian monsoon regions (Fig. 2d). Although there are the similarities over Asia and southwestern North America between Figs 1a and 2d, a larger difference appears over extratropical North America. This indicates that the North American land surface heating anomaly tends to force an in-phase relationship of precipitation between Asian monsoon regions and extratropical North America. It is clear that the change of the North American land surface heating cannot force the observed CANA pattern and the observed positive SAT-negative precipitation anomaly relationship over extratropical North America. Compared to the Eurasian land surface heating anomaly, therefore, the land heating anomaly over North America plays a minor role in forcing the CANA precipitation pattern.

Because the coupled CCSM3 model includes ocean-atmosphere interactions, we further examine the results from the NCAR’s Community Atmosphere Model version 3 (CAM3) with prescribed climatological mean SST (without ocean-atmosphere interactions). The forced anomalies in Figs 2a,b and 3a are also generally obtained in the CAM3 simulations with the land surface heating anomaly over Europe and West Asia (Figs 2e,f and 3c), although the positive and negative anomalies of SAT and precipitation in the CAM3 simulations are relatively weak over Asia and North America. This indicates that the ocean-atmosphere interactions may amplify the magnitude of the response to the Europe-West Asia surface thermal forcing.

Figure 1f and S9 further show the regressions of SST against the PC1 of the CANA precipitation pattern. SST anomalies are generally weak (within 0.2 °C) before April, with weak positive SST anomalies in the tropical Pacific (Fig. S9). Starting from April, negative SST anomalies develop along the equatorial eastern Pacific with the largest negative value in excess of 0.5 °C, and expand to the west of 180° in summer. This indicates that the summer CANA pattern is associated with the development of a La Nina state from spring to summer.

Previous studies showed that the ENSO causes episodes of warm-season extreme rainfall anomalies in Asia and North America over a wide range of time scales^6^,^7^,^8^. We further examine the impact of the Eurasian non-monsoon heating and equatorial central-eastern Pacific cooling anomalies individually and collaboratively. In the CCSM3 simulation (with the coupled atmosphere and ocean model) of reducing AMJJA surface vegetation albedo in Europe and West Asia, the equatorial central-eastern Pacific cooling anomalies is nudged toward the model SST (Fig. S10a) (see Methods for details). Figure 2g,h shows the responses of AMJJA mean SAT and JJA precipitation to this collaborative forcing in the CCSM3 model. Generally speaking, the features similar to Fig. 2a,b are forced by both the land and ocean heating anomalies. Large positive SAT anomalies remarkably move northward over Europe and eastward over extratropical North America, and SAT anomalies reduce over central Asia and central-eastern Russia and increase over the eastern coasts of Russia (Fig. 2g). Meanwhile, precipitation anomalies increase over central-eastern Russia and the tropics of South America (Fig. 2h). These features are more consistent to the observations, further indicating a positive contribution of the ENSO to the forcing of the Eurasian land heating anomaly. However, the equatorial central-eastern Pacific cooling alone (Fig. S10b) mainly results in positive precipitation anomalies over South and Southeast Asia and South America and to the south of 40°N over North America (generally not significant) and negative rainfall anomalies in a small area of extratropical North America (Fig. 2j), instead of forcing a remarkable CANA precipitation pattern. In addition, it does not cause significant positive SAT anomalies over Europe, West Asia, and extratropical North America (Fig. 2i) and large SST anomalies over the extratropical Pacific and Atlantic (Fig. S10b).

## Concluding Remarks

Traditional theory has attributed large-scale precipitation anomalies to changes in tropical oceans, such as ENSO. But our result clearly shows that the ENSO impacts alone tend to be confined in the tropics during northern summer. The forcing impact of land surface heating over the Eurasian non-monsoon regions plays a dominant role in the determinations of the summer Asian and North American precipitation and North Pacific and Atlantic anticyclonic anomalies outside of the tropics. Compared to the Eurasian land heating, the impact of North American land heating is minor. Meanwhile, the feedbacks of global ocean-atmosphere interactions add a positive contribution to the forcing of the Eurasian land heating, especially the Eurasian land heating anomaly and the ENSO exhibit a collaborative impact. But this positive contribution from the ENSO is secondary. These findings provide new insight to the origin of summer precipitation variations in Asia and North America. Moreover, it is noted that large negative SAT anomalies appear in East Asia between 35°N and 60°N, indicating the cooling East Asian land. This region has been identified as a hot spot of land-atmosphere coupling effects on summer climate^14^. Thus it will be instructive to examine a potential influence of the cooling East Asian land on the summer CANA precipitation anomaly pattern in the future work.

The result here adds a new perspective for further mining the sources of the precipitation predictability over Asia and North America. In particular, it advances our understanding of the impact of global warming on regional hydrological cycle. Under the projected global warming due to the anthropogenic forcing, the prominent surface warming over Eurasian non-monsoon regions is a robust feature^23^ which, through the mechanism discussed here, would favor an increase of precipitation in the Asian monsoon regions and a decrease of precipitation in the mid- and high-latitude North America. This is indeed the case as projected by the CMIP5 models^5^. Therefore, looking for new climate signals over land will be a challenge to persistently reduce the uncertainties of precipitation predication over Asia and North America under the global warming.

## Methods

We utilize SAT and precipitation data during 1901–2009 from the CRU analysis^24^, the 1901–2009 tropospheric temperature, geopotential height, winds, specific humidity, and vertical velocity from the twentieth century reanalysis V2 products^25^, and the 1979–2013 Global Precipitation Climatology Project (GPCP) precipitation^26^, in which the linear trend of SAT is removed to reduce its effect on correlation coefficients. Moreover, the 1901–2009 monthly mean SST from the Hadley Centre Sea Ice and Sea Surface Temperature (HadISST) data^27^ is also utilized.

We use the 1901–1999 NCAR’s PCM model data from the IPCC simulations for the 20th century (20C3M) of the Fourth Assessment^28^. This model consists of the CAM3 model, the Land Surface Model, the DOE Los Alamos National Laboratory Parallel Ocean Program (POP) ocean model, and the Community Sea Ice Model. The PCM can reasonably well reproduce the Asian summer monsoon circulation intensity and its relationship with the tropospheric land-sea thermal contrasts^29^, and the CCSM3 can well capture the variations of extratropical tropospheric atmospheric circulation over the Northern Hemisphere associated with the ENSO during summer^30^.

Reduction of land surface albedo can increase SAT in climate models, which has been used to understand the impacts of strengthened land surface heating on atmospheric circulation^9^,^11^,^13^,^21^,^31^. Here, three experiments with the NCAR’s atmosphere-ocean coupled CCSM3 (with the same atmospheric, ocean, and sea ice components as those of PCM, and the Community Land Model) are conducted to understand impacts of land heating over Eurasia or North America. The first experiment is an unforced experiment (the control experiment), which is the same as the CCSM3 coupled general circulation model experiment downloaded from the NCAR’s website, called the experiment CCSM3_C. Referring to the positions of positive SAT anomalies in both Fig. 1e and S4b, we conduct the second experiment that is the same as the experiment CCSM3_C but with a change in vegetation over Europe and West Asia (25°N-60°N, 0°-80°E) (a non-monsoon region^4^,^5^) (see the box shown in Fig. 1e), called the experiment CCSM3_EA. In this experiment, the surface vegetation type at each grid of the specified Eurasian region is prescribed as the needle leaf evergreen temperate tree from the original type of grass, or shrub, or bare soil in the experiment CCSM3_C from April to August. As the result of changing the vegetation, there is a lower albedo value for long-/short-wave radiation in the experiment CCSM3_EA relative to the experiment CCSM3_C. Figure S11a-d shows the albedo difference of long-/short-wave direct or scattered radiation between the experiments CCSM3_EA and CCSM3_C. The reduced albedo allows more incoming radiation to be absorbed by the land surface, which in turn enhances the local land surface heating to the atmosphere. The third experiment is the same as the experiment CCSM3_EA but with a change in surface vegetation over the North American non-monsoon region (30°N-60°N, 125°W-65°W) (Fig. 1e and S11e-h), called the experiment CCSM3_NA. Compared to Fig. S3, this warming over North America, especially in May, is amplified to some extent. For each experiment, the model is integrated for 70 years and the mean values over the last 20 years are used in the analysis. Since the prescribed boundary conditions repeat annually, but the atmospheric initial conditions vary, each year can be considered as an independent realization (ensemble member). To further separate effects of the Europe-West Asia land heating from those of ocean-atmosphere interactions in the coupled CCSM3, using the atmospheric component of the CCSM3 coupled model (the CAM3), we conduct two similar experiments, called the experiments CAM3_C and CAM3_EA, in which the CAM3 is integrated for 30 years under the condition of prescribed climatological mean SST. The mean values over the last 20 years are analyzed.

To examine a potential influence of the equatorial central-eastern Pacific cooling alone in the coupled atmosphere and ocean CCSM3 model, we refer to the positions of the observed equatorial Pacific SST anomalies (Fig. S9), follow the SST nudging method of Rosati *et al.*^32^, and nudge positive SST anomalies toward the coupled model SST over 10°S–10°N/180°W–80°W from April to August. In this experiment (called the experiment CCSM3_P), the CCSM3 is integrated for 8 months from January 1. We repeat this experiment twenty times with different initial conditions that come from January 1 of the 20 model years in the experiment CCSM3_C, and analyze an average of twenty ensemble experiments. Figure S10b shows the constructed negative differences (CCSM3_C minus CCSM3_P) with a negative central value in excess of 1 °C in the tropical central and eastern Pacific. This feature is similar to the observation (Fig. 1f), which demonstrates the reasonability of the experiment CCSM3_P. Another experiment is used to examine a collaborative influence of the Europe-West Asia land heating and equatorial central-eastern Pacific cooling anomalies, called the experiment CCSM3_EAP, in which the methods of changing the Eurasian land heating and Pacific cooling are consistent with the experiments CCSM3_EA and CCSM3_P (but with negative SST anomalies), respectively. Negative SST anomalies with a central value of −1 °C are constructed in the tropical central and eastern Pacific (Fig. S10a).

## Additional Information

**How to cite this article**: Zhao, P. *et al.* Summer precipitation anomalies in Asia and North America induced by Eurasian non-monsoon land heating versus ENSO. *Sci. Rep.* **6**, 21346; doi: 10.1038/srep21346 (2016).

## Supplementary Material

Supplementary Information
