# Supplementary material for: Summer precipitation anomalies in Asia and North America induced by Eurasian non-monsoon land heating versus ENSO
Source: Sci Rep. 2016 Feb 26;6:21346. doi: 10.1038/srep21346 (PMC4768350; doi:10.1038/srep21346)
Supplement: Supplementary Information [file srep21346-s1.pdf]

Summer precipitation anomalies in Asia and North America induced by Eurasian non-monsoon land heating versus ENSO

Ping Zhao[1,2], Bin Wang[3,4], Jiping Liu[5], Xiuji Zhou[1], Junming Chen[1], Sulan Nan[1], Ge Liu[1], Dong Xiao[1]

[1]State Key Laboratory of Severe Weather, Chinese Academy of Meteorological Sciences, Beijing, China;

[2]Collaborative Innovation Center on Forecast and Evaluation of Meteorological Disasters, Nanjing University of Information Science and Technology, Nanjing, China;

[3]International Pacific Research Center, School of Ocean and Earth Science and Technology, University of Hawaii at Manoa, Honolulu, HI, USA;

[4]Earth System Modeling Center, Nanjing University of Information Science and Technology, Nanjing, China.

[5]Department of Atmospheric and Environmental Sciences, University at Albany, State University of New York, Albany, NY, USA;

Corresponding author: zhaoping@camscma.cn

Supplementary Figure Captions

Figure S1. (a) JJA mean GPCP precipitation anomaly (from the 1979-2013 mean) in 2012; (b) multi-model mean changes in JJA mean precipitation (mm day^{-1}) for the RCP 4.5 scenario for the period 2070–2095 relative to historical simulation for the period 1980–2005 in CMIP5[5], in which red and grey contours are for monsoon and dry domains in turn; and (c) the 40-yr running correlation coefficient of JJA CRU precipitation between Asia (70°E-120°E, 10°N-60°N) and the Great Plains (115°W-95°W, 35°N-45°N) of North America during 1901–2009, in which the number 1 in the abscissa is for the correlation during 1901–40, 2 is for the correlation during 1902–41, . . . , up to 70 for the correlation during 1970–2009, and the dot, long, and long-short lines are significant for the 90%, 95%, and 98% confidence levels in turn. Fig. 1a and c is generated by Grid Analysis and Display System (GrADS) Version 2.0.1.oga.1 with Copyright (c) 1988-2011 by Brian Doty and the Institute for Global Environment and Society (IGES) (ftp://cola.gmu.edu/grads/2.0/old/). Fig. 1b comes from the published reference (without any modification) and we have gotten the permission of citing this figure from the corresponding author of this reference (Bin Wang, also the second author of our paper).

Figure S2. Regression of JJA mean 700-hPa water vapor flux ($q\boldsymbol{V}$) of the twentieth century reanalysis V2 products against the PC1 during 1901-2009, in which q is specific humidity, $\boldsymbol{V}$ is the horizontal wind vector, and the grey shaded area is for topography. This figure is generated by Grid Analysis and Display System (GrADS) Version 2.0.1.oga.1 with Copyright (c) 1988-2011 by Brian Doty and the Institute for Global Environment and Society (IGES) (ftp://cola.gmu.edu/grads/2.0/old/).

Figure S3. Regression of monthly mean CRU surface air temperature against the PC1

during 1901-2009, in which the two boxes indicate subtropical and midlatitude Eurasia and North America in turn. These figures are generated by Grid Analysis and Display System (GrADS) Version 2.0.1.oga.1 with Copyright (c) 1988-2011 by Brian Doty and the Institute for Global Environment and Society (IGES) (ftp://cola.gmu.edu/grads/2.0/old/).

Figure S4. (a) JJA mean precipitation EOF1 mode (PC1) of the NCAR's PCM 20C3M simulation during 1901-1999; and (b) regression of AMJJA model surface air temperature against the model PC1 during 1901-1999. These figures are generated by Grid Analysis and Display System (GrADS) Version 2.0.1.oga.1 with Copyright (c) 1988-2011 by Brian Doty and the Institute for Global Environment and Society (IGES) (ftp://cola.gmu.edu/grads/2.0/old/).

Figure S5. (a) Response of AMJJA mean sensible heat flux (W m^{-2}) at the surface to the Eurasian land surface thermal forcing in the coupled CCSM3 model (CCSM3_EA minus CCSM3_C); and (b) same as in (a) but for latent heat flux. Black dot is at the 90% confidence level. These figures are generated by Grid Analysis and Display System (GrADS) Version 2.0.1.oga.1 with Copyright (c) 1988-2011 by Brian Doty and the Institute for Global Environment and Society (IGES) (ftp://cola.gmu.edu/grads/2.0/old/).

Figure S6. (a) Longitude-height cross section of the climatological mean JJA eddy temperature (T'; unit: °C; shaded) and geopotential height (H'; unit: ×10 m; contour) from the twentieth century reanalysis V2 products along 35°N (black shaded areas denote mountains), in which the eddy variable is defined as the difference between the variable and its global zonal mean; and (b) same as in (a) but for 200-hPa flow and 500-200-hPa mean T' (°C; shaded). These figures are generated by Grid Analysis and Display System (GrADS) Version 2.0.1.oga.1 with

Copyright (c) 1988-2011 by Brian Doty and the Institute for Global Environment and Society (IGES) (ftp://cola.gmu.edu/grads/2.0/old/).

Figure S7. (a) Longitude-height cross section of responses of JJA mean T' (°C; shaded) and H' (m; contour) to the Eurasian land surface thermal forcing in the coupled CCSM3 model along 30°-50°N; (b) same as in (a) but for the North American land surface thermal forcing; and (c) same as in (a) but for regressions of JJA mean T' and H' against the PC1 in observation during 1901-2009. These figures are generated by Grid Analysis and Display System (GrADS) Version 2.0.1.oga.1 with Copyright (c) 1988-2011 by Brian Doty and the Institute for Global Environment and Society (IGES) (ftp://cola.gmu.edu/grads/2.0/old/).

Figure S8. (a) Response of JJA mean *p*-velocity (0.001×Pa s^{-1}) to the Eurasian land surface thermal forcing in the coupled CCSM3 model along 30°-50°N; (b) same as in (a) but for the North American land surface thermal forcing; and (c) same as in (a) but for regression of JJA mean *p*-velocity (0.01×Pa s^{-1}) against the PC1 in observation during 1901-2009. These figures are generated by Grid Analysis and Display System (GrADS) Version 2.0.1.oga.1 with Copyright (c) 1988-2011 by Brian Doty and the Institute for Global Environment and Society (IGES) (ftp://cola.gmu.edu/grads/2.0/old/).

Figure S9. Regression of monthly mean HadISST SST against the PC1 in observation during 1901-2009, in which the box indicates the equatorial central-eastern Pacific. These figures are generated by Grid Analysis and Display System (GrADS) Version 2.0.1.oga.1 with Copyright (c) 1988-2011 by Brian Doty and the Institute for Global Environment and Society (IGES) (ftp://cola.gmu.edu/grads/2.0/old/).

Figure S10. (a) Difference of AMJJA mean SST between the sensitivity experiment with both Eurasian land heating and equatorial central-eastern Pacific cooling

anomalies and the control experiment in the coupled atmosphere and ocean CCSM3 model, in which the box indicates the equatorial central-eastern Pacific; and (b) same as in (a) but for the difference between the control experiment and the sensitivity experiment with the equatorial central-eastern Pacific warming anomaly alone. These figures are generated by Grid Analysis and Display System (GrADS) Version 2.0.1.oga.1 with Copyright (c) 1988-2011 by Brian Doty and the Institute for Global Environment and Society (IGES) (ftp://cola.gmu.edu/grads/2.0/old/).

Figure S11. Differences of albedo between the experiment with a change of vegetation types in Eurasia or America and the control experiment. For long-wave direct (a) and scattered (b) radiations and short-wave direct (c) and scattered (d) radiations over Eurasia; and (e)-(h) same as in (a)-(d) but over North America. These figures are generated by Grid Analysis and Display System (GrADS) Version 2.0.1.oga.1 with Copyright (c) 1988-2011 by Brian Doty and the Institute for Global Environment and Society (IGES) (ftp://cola.gmu.edu/grads/2.0/old/).

Supplementary Figures

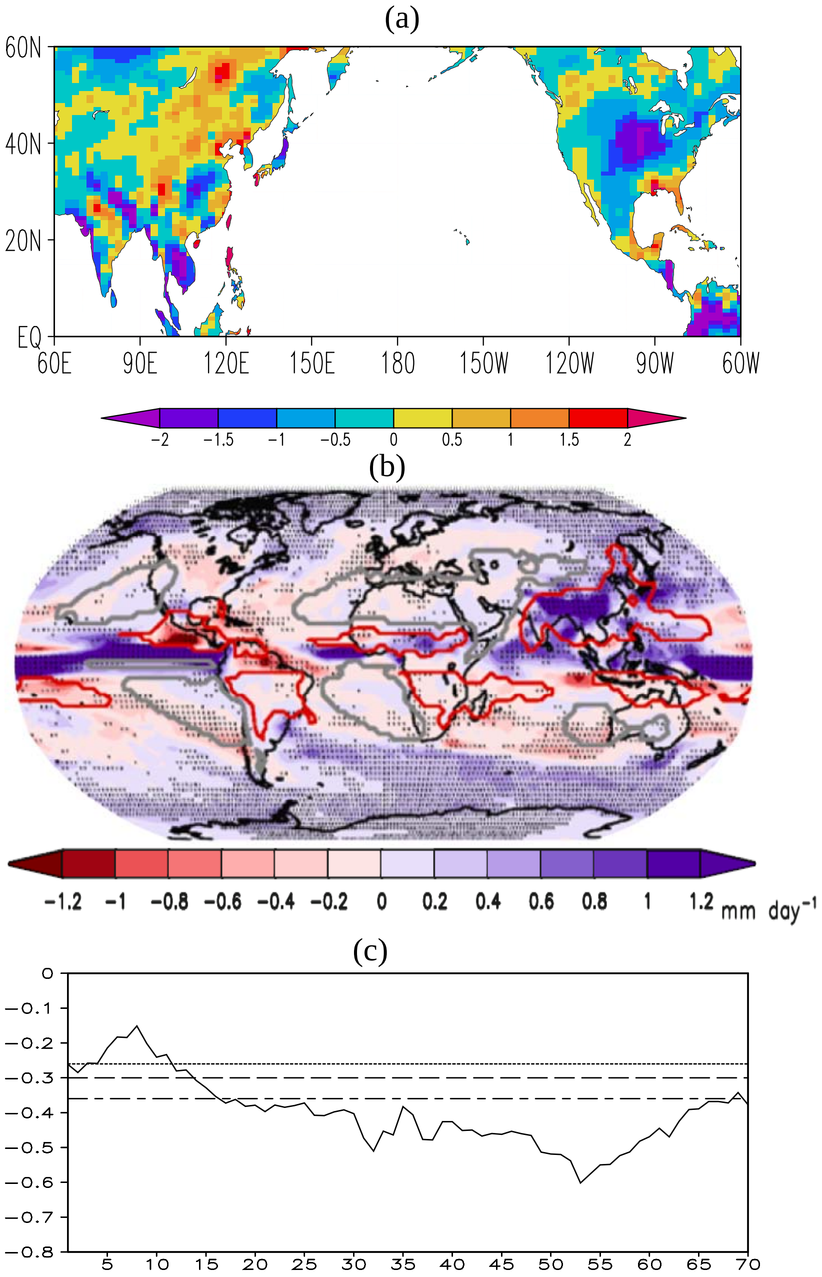

Figure S1. (a) JJA mean GPCP precipitation anomaly (from the 1979-2013 mean) in 2012; (b) multi-model mean changes in JJA mean precipitation (mm day^{-1}) for the RCP 4.5 scenario for the period 2070–2095 relative to historical simulation for the period 1980–2005 in CMIP5[5], in which red and grey contours are for monsoon and dry domains in turn; and (c) the 40-yr running correlation coefficient of JJA CRU precipitation between Asia (70°E-120°E, 10°N-60°N) and the Great Plains

(115°W-95°W, 35°N-45°N) of North America during 1901–2009, in which the number 1 in the abscissa is for the correlation during 1901–40, 2 is for the correlation during 1902–41, . . . , up to 70 for the correlation during 1970–2009, and the dot, long, and long-short lines are significant for the 90%, 95%, and 98% confidence levels in turn. Fig. 1a and c is generated by Grid Analysis and Display System (GrADS) Version 2.0.1.oga.1 with Copyright (c) 1988-2011 by Brian Doty and the Institute for Global Environment and Society (IGES) (ftp://cola.gmu.edu/grads/2.0/old/). Fig. 1b comes from the published reference (without any modification) and we have gotten the permission of citing this figure from the corresponding author of this reference (Bin Wang, also the second author of our paper).

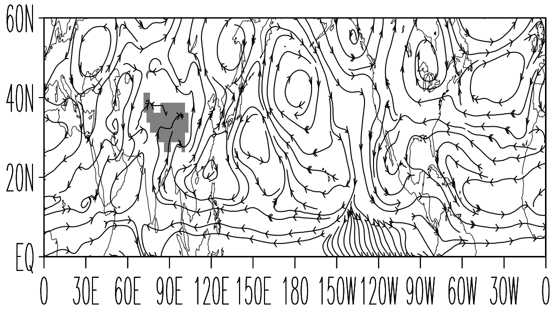

Figure S2. Regression of JJA mean 700-hPa water vapor flux ($q\boldsymbol{V}$) of the twentieth century reanalysis V2 products against the PC1 during 1901-2009, in which q is specific humidity, $\boldsymbol{V}$ is the horizontal wind vector, and the grey shaded area is for topography. This figure is generated by Grid Analysis and Display System (GrADS) Version 2.0.1.oga.1 with Copyright (c) 1988-2011 by Brian Doty and the Institute for Global Environment and Society (IGES) (ftp://cola.gmu.edu/grads/2.0/old/).

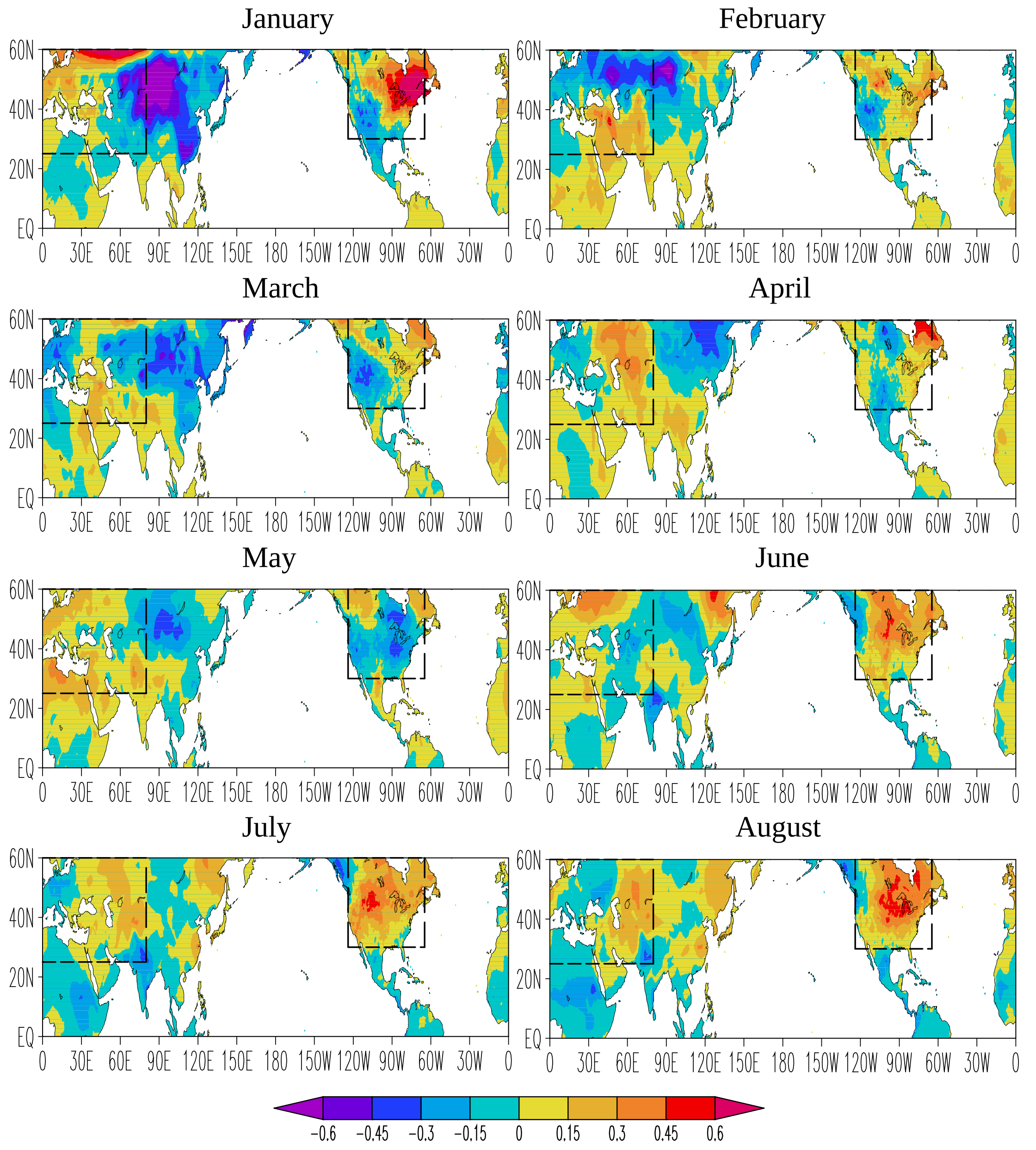

Figure S3. Regression of monthly mean CRU surface air temperature against the PC1 during 1901-2009, in which the two boxes indicate subtropical and midlatitude Eurasia and North America in turn. These figures are generated by Grid Analysis and Display System (GrADS) Version 2.0.1.oga.1 with Copyright (c) 1988-2011 by Brian Doty and the Institute for Global Environment and Society (IGES) (ftp://cola.gmu.edu/grads/2.0/old/).

(a) (b)

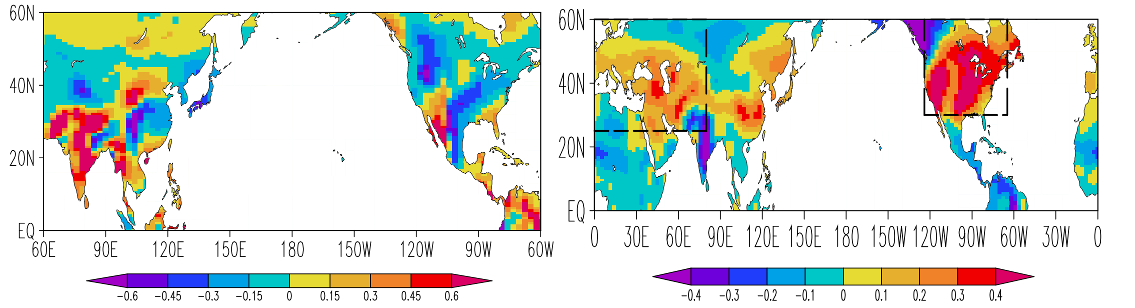

Figure S4. (a) JJA mean precipitation EOF1 mode (PC1) of the NCAR's PCM 20C3M simulation during 1901-1999; and (b) regression of AMJJA model surface air temperature against the model PC1 during 1901-1999. These figures are generated by Grid Analysis and Display System (GrADS) Version 2.0.1.oga.1 with Copyright (c) 1988-2011 by Brian Doty and the Institute for Global Environment and Society (IGES) (ftp://cola.gmu.edu/grads/2.0/old/).

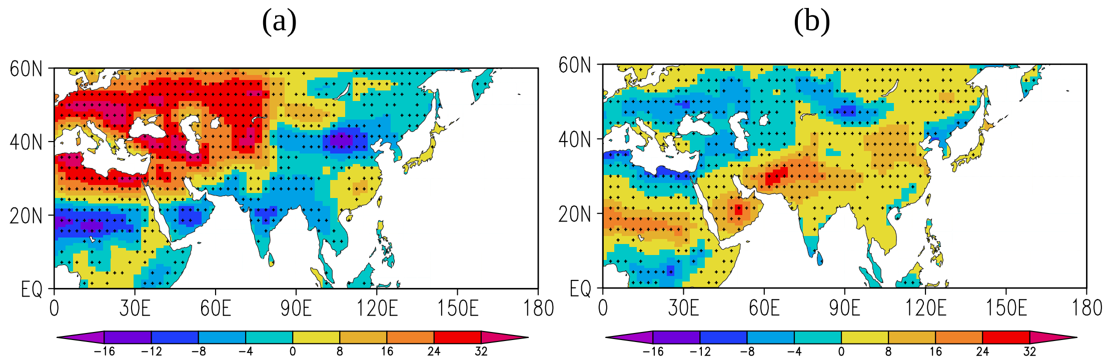

Figure S5. (a) Response of AMJJA mean sensible heat flux (W m^{-2}) at the surface to the Eurasian land surface thermal forcing in the coupled CCSM3 model (CCSM3_EA minus CCSM3_C); and (b) same as in (a) but for latent heat flux. Black dot is at the 90% confidence level. These figures are generated by Grid Analysis and Display System (GrADS) Version 2.0.1.oga.1 with Copyright (c) 1988-2011 by Brian Doty and the Institute for Global Environment and Society (IGES) (ftp://cola.gmu.edu/grads/2.0/old/).

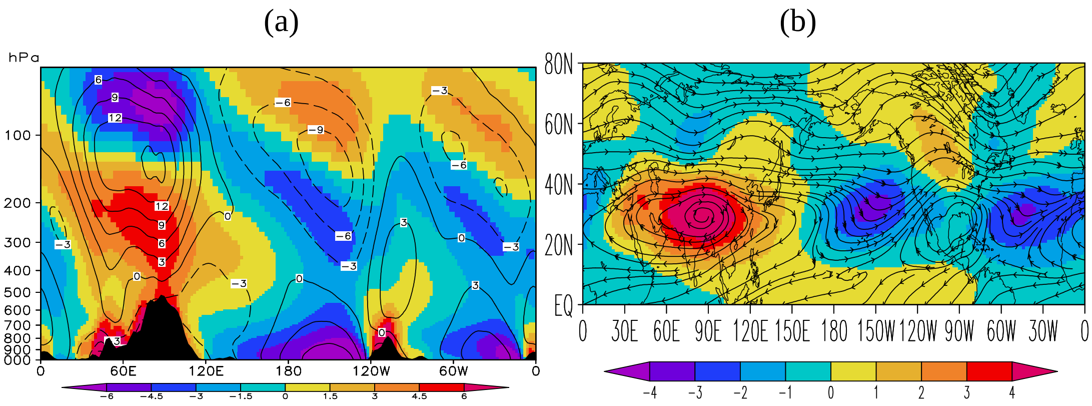

Figure S6. (a) Longitude-height cross section of the climatological mean JJA eddy temperature (T'; unit: °C; shaded) and geopotential height (H'; unit: ×10 m; contour) from the twentieth century reanalysis V2 products along 35°N (black shaded areas denote mountains), in which the eddy variable is defined as the difference between the variable and its global zonal mean; and (b) same as in (a) but for 200-hPa flow and 500-200-hPa mean T' (°C; shaded). These figures are generated by Grid Analysis and Display System (GrADS) Version 2.0.1.oga.1 with Copyright (c) 1988-2011 by Brian Doty and the Institute for Global Environment and Society (IGES) (ftp://cola.gmu.edu/grads/2.0/old/).

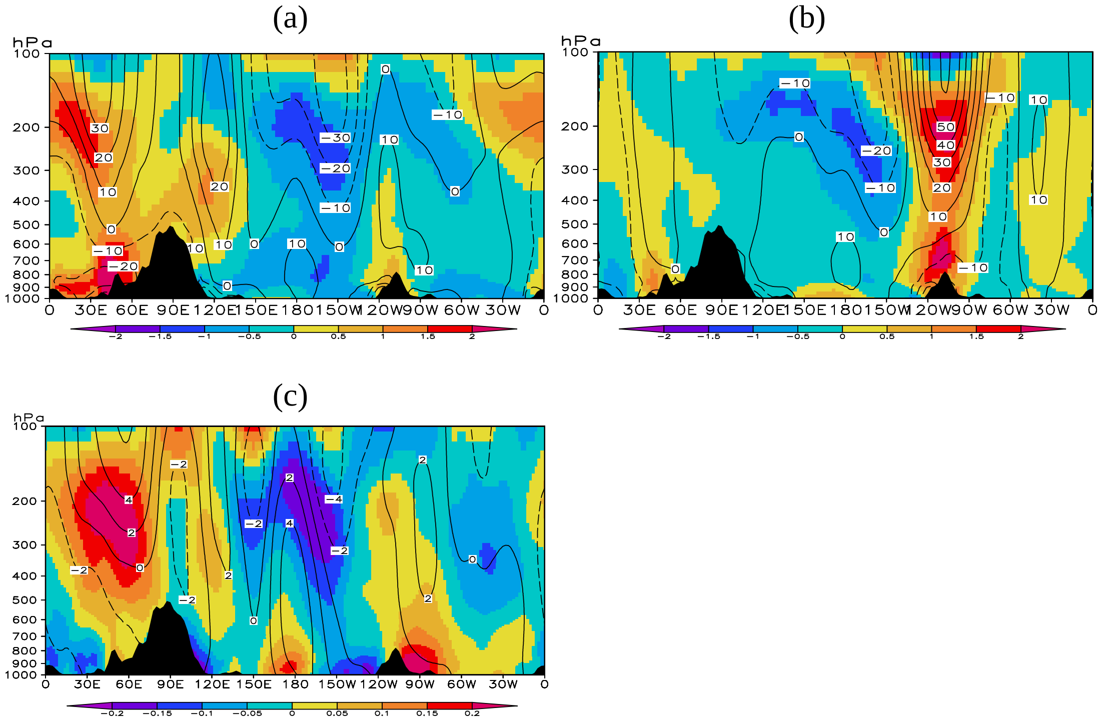

Figure S7. (a) Longitude-height cross section of responses of JJA mean T' (°C; shaded) and H' (m; contour) to the Eurasian land surface thermal forcing in the coupled CCSM3 model along 30°-50°N; (b) same as in (a) but for the North American land surface thermal forcing; and (c) same as in (a) but for regressions of JJA mean T' and H' against the PC1 in observation during 1901-2009. These figures are generated by Grid Analysis and Display System (GrADS) Version 2.0.1.oga.1 with Copyright (c) 1988-2011 by Brian Doty and the Institute for Global Environment and Society (IGES) (ftp://cola.gmu.edu/grads/2.0/old/).

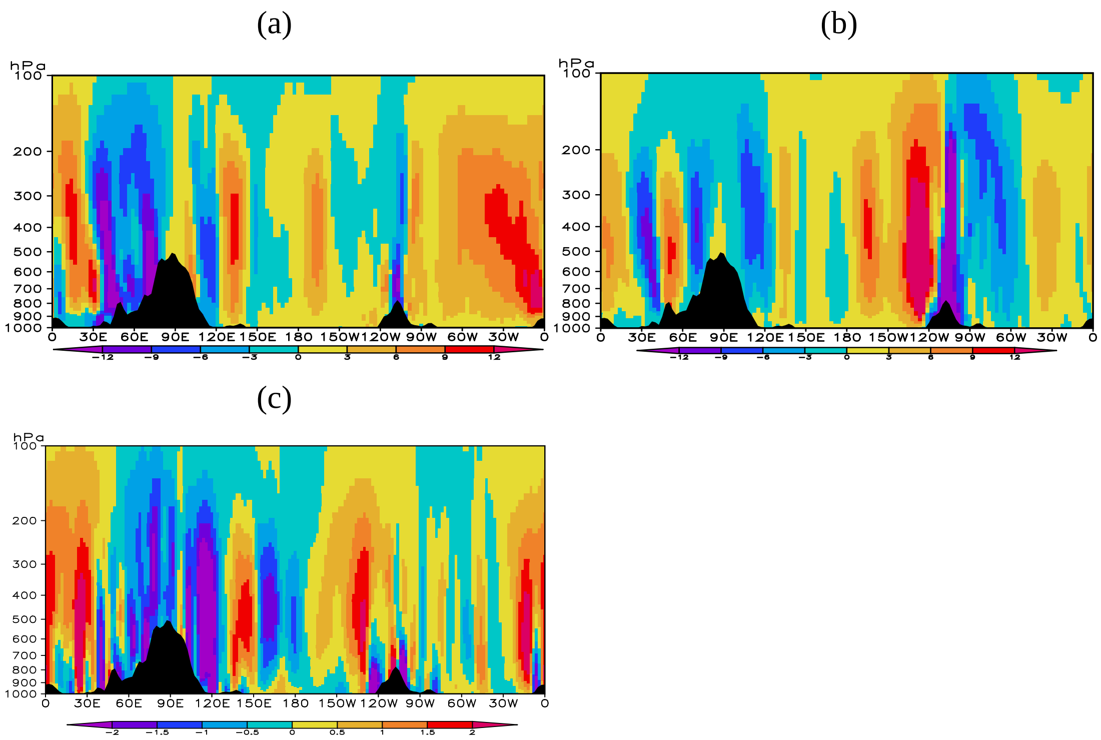

Figure S8. (a) Response of JJA mean *p*-velocity ($0.001\times$Pa s^{-1}) to the Eurasian land surface thermal forcing in the coupled CCSM3 model along 30°-50°N; (b) same as in (a) but for the North American land surface thermal forcing; and (c) same as in (a) but for regression of JJA mean *p*-velocity ($0.01\times$Pa s^{-1}) against the PC1 in observation during 1901-2009. These figures are generated by Grid Analysis and Display System (GrADS) Version 2.0.1.oga.1 with Copyright (c) 1988-2011 by Brian Doty and the Institute for Global Environment and Society (IGES) (ftp://cola.gmu.edu/grads/2.0/old/).

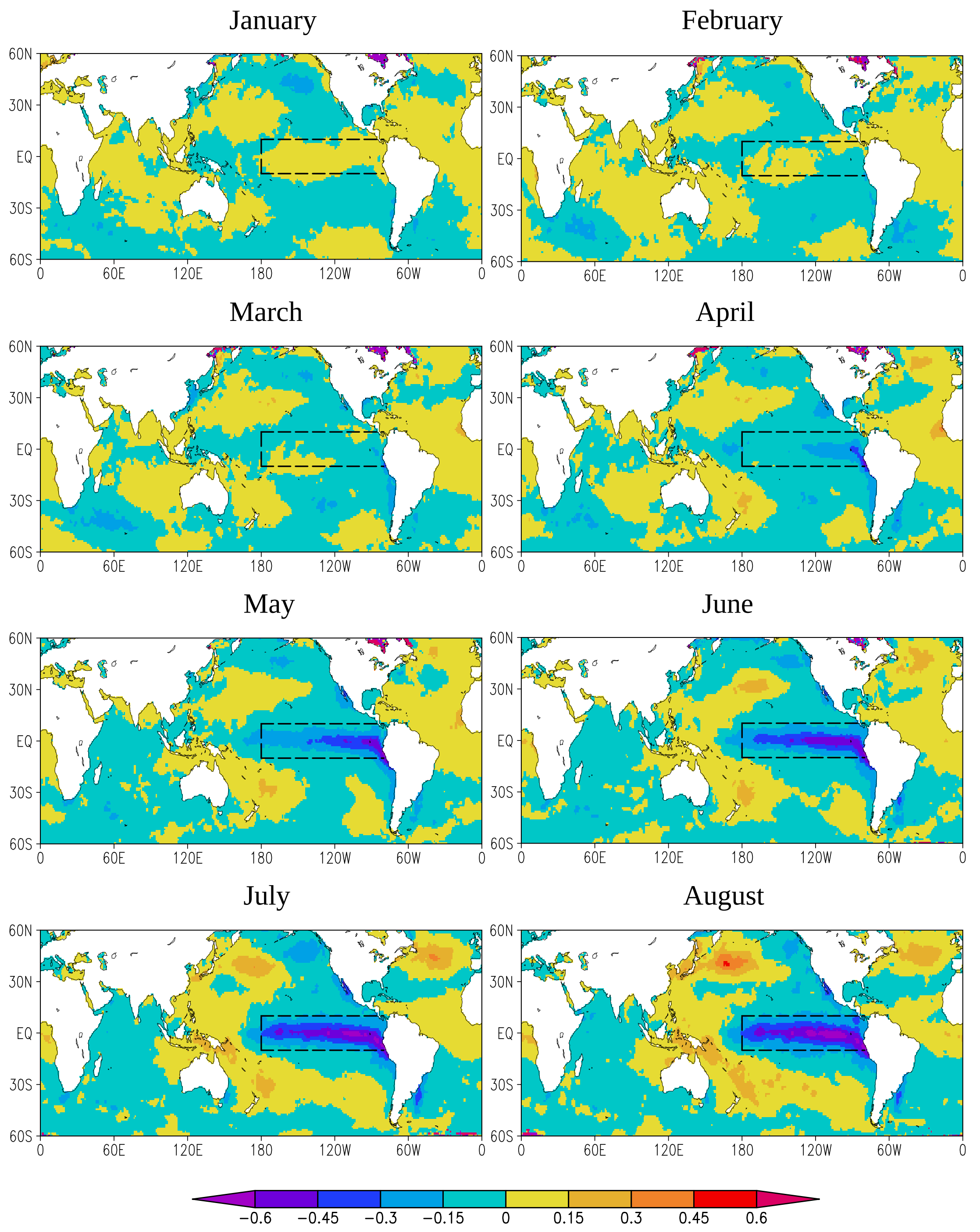

Figure S9. Regression of monthly mean HadISST SST against the PC1 in observation during 1901-2009, in which the box indicates the equatorial central-eastern Pacific. These figures are generated by Grid Analysis and Display System (GrADS) Version 2.0.1.oga.1 with Copyright (c) 1988-2011 by Brian Doty and the Institute for Global Environment and Society (IGES) (ftp://cola.gmu.edu/grads/2.0/old/).

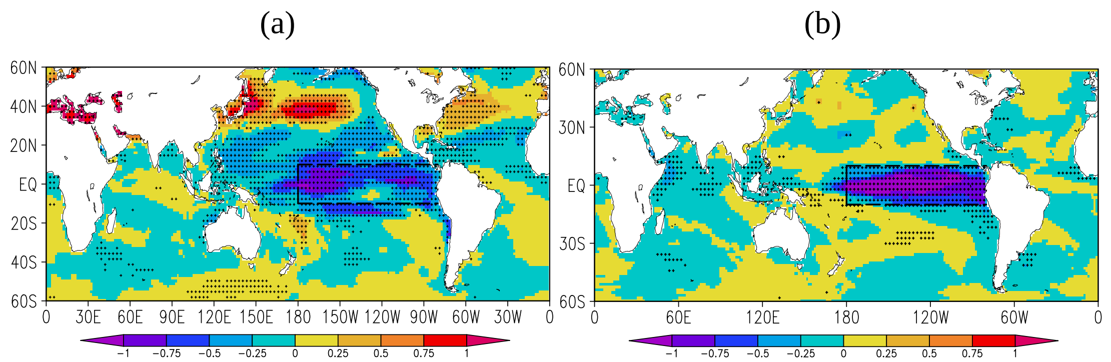

Figure S10. (a) Difference of AMJJA mean SST between the sensitivity experiment with both Eurasian land heating and equatorial central-eastern Pacific cooling anomalies and the control experiment in the coupled atmosphere and ocean CCSM3 model, in which the box indicates the equatorial central-eastern Pacific; and (b) same as in (a) but for the difference between the control experiment and the sensitivity experiment with the equatorial central-eastern Pacific warming anomaly alone. These figures are generated by Grid Analysis and Display System (GrADS) Version 2.0.1.oga.1 with Copyright (c) 1988-2011 by Brian Doty and the Institute for Global Environment and Society (IGES) (ftp://cola.gmu.edu/grads/2.0/old/).

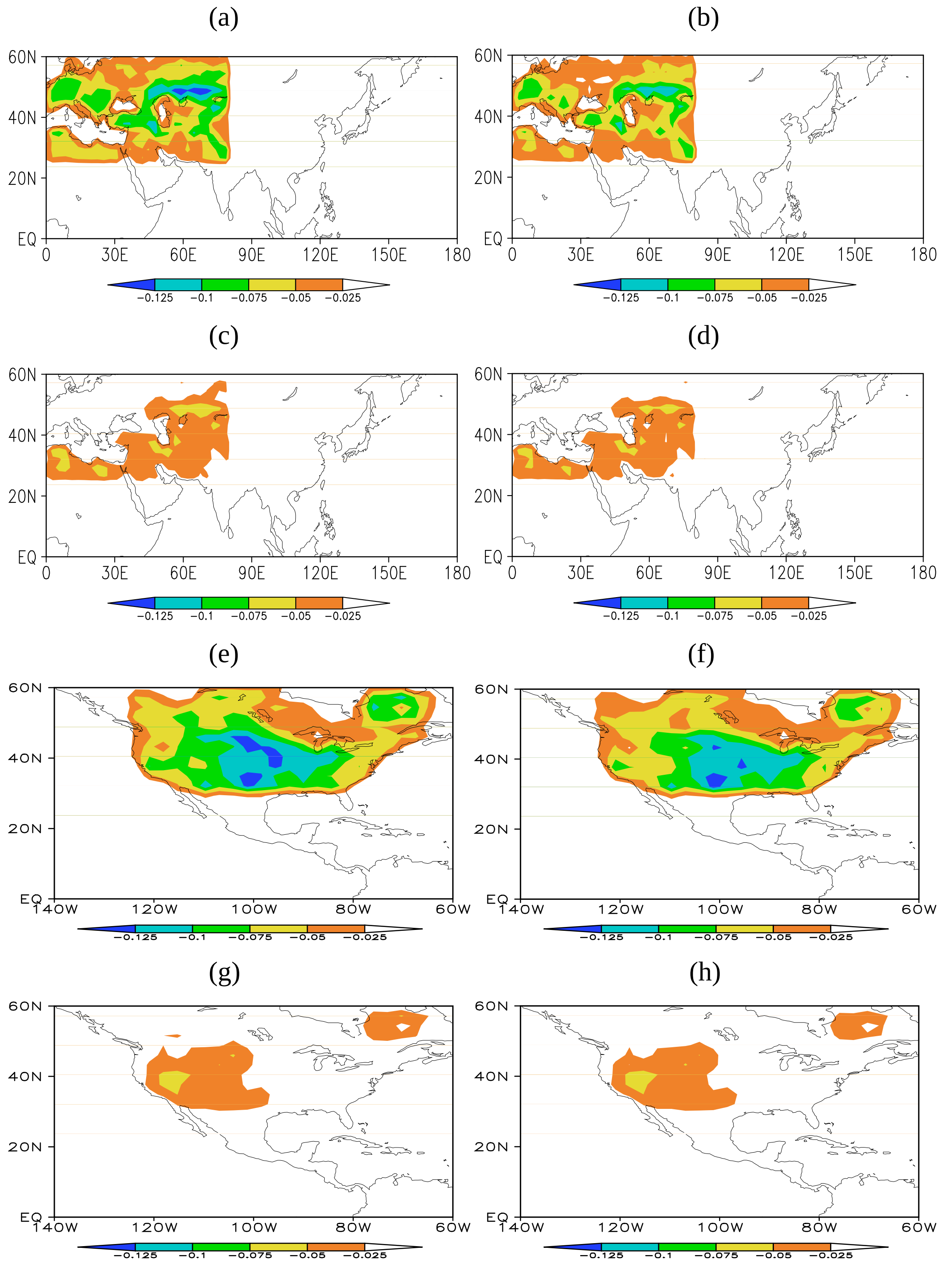

Figure S11. Differences of albedo between the experiment with a change of vegetation types in Eurasia or America and the control experiment. For long-wave direct (a) and scattered (b) radiations and short-wave direct (c) and scattered (d) radiations over Eurasia; and (e)-(h) same as in (a)-(d) but over North America. These figures are generated by Grid Analysis and Display System (GrADS) Version

2.0.1.oga.1 with Copyright (c) 1988-2011 by Brian Doty and the Institute for Global Environment and Society (IGES) (ftp://cola.gmu.edu/grads/2.0/old/).
